# Case Report: Autoimmune-associated tubulointerstitial nephritis with predominant Sjögren features in a child presenting with recurrent sterile pyuria

**DOI:** 10.3389/fped.2026.1833551

**Published:** 2026-08-20

**Authors:** Tianyuan Han, Hong Chang, Nana Nie, Jia Liu, Ranran Zhang, Yi Lin, Cui Bai, Shan Gao, Qiuye Zhang, Dahai Wang

**Affiliations:** Department of Pediatric Nephrology, Rheumatology and Immunology, The Affiliated Hospital of Qingdao University, Qingdao, Shandong, China

**Keywords:** autoimmune diseases, interstitial nephritis, Sjögren syndrome, sterile pyuria, systemic lupus erythematosus

## Abstract

Tubulointerstitial nephritis (TIN) is an important cause of renal tubular dysfunction in children but may be misdiagnosed as urinary tract infection (UTI) because of non-specific urinary manifestations. Autoimmune-associated TIN is particularly challenging when overlapping autoimmune features are present. Here, we report a pediatric case of TIN with an autoimmune-associated phenotype initially presenting as recurrent culture-negative urinary symptoms suggestive of UTI. A 5-year-old boy presented with recurrent urinary frequency, persistent pyuria, and negative urine cultures. Renal ultrasonography revealed hydronephrosis and bladder abnormalities, while renal scintigraphy suggested pyelonephritis. Despite antibiotic therapy, the urinary abnormalities persisted. Further evaluation showed polyuria, polydipsia, and hyposthenuria, indicating tubular concentrating impairment. A renal biopsy demonstrated severe tubulointerstitial inflammation with tubular injury, mild glomerular changes, negative immunofluorescence, and absence of electron-dense deposits. An immunological evaluation revealed high-titer antinuclear antibodies, anti-double-stranded DNA antibody positivity, elevated rheumatoid factor, and hypergammaglobulinemia, whereas the results of anti-Sjögren syndrome–related antigen A antibody and anti-Sjögren syndrome-–related antigen B antibody tests were negative. Salivary gland abnormalities were identified by ultrasonography, while a labial biopsy showed chronic inflammation without diagnostic focal lymphocytic sialadenitis. The patient did not fulfill the 2016 ACR/EULAR classification criteria for Sjögren syndrome, and systemic lupus erythematosus remained insufficiently supported as a definitive diagnosis. Based on comprehensive evaluation, the case was considered autoimmune-associated TIN with predominant Sjögren features. Glucocorticoid and immunosuppressive therapy led to the resolution of urinary abnormalities. This case highlights the need to consider autoimmune-associated TIN in children with recurrent culture-negative urinary symptoms and persistent pyuria.

## 1 Introduction

Tubulointerstitial nephritis (TIN) is an inflammatory disease primarily affecting the renal tubules and interstitium, with diverse clinical manifestations. Common urinary abnormalities observed in TIN include renal tubular acidosis, leukocyturia, proteinuria, hematuria, glycosuria, hypokalemia, and impaired urinary concentrating ability. Renal biopsy is the gold standard for the diagnosis of TIN, with typical pathological features such as interstitial edema, inflammatory cell infiltration, and tubular injury, whereas the glomeruli and blood vessels are usually only mildly involved (1–3). Given that the clinical manifestations of TIN are non-specific and renal biopsy is often delayed because of its invasive nature and associated risks, misdiagnosis or underdiagnosis is common. In particular, when leukocyturia is the sole manifestation, TIN may be misdiagnosed as a urinary tract infection (UTI). TIN has heterogeneous etiologies, including drug-related, infectious, autoimmune, and systemic inflammatory causes. Autoimmune diseases such as Sjögren syndrome (SS) and systemic lupus erythematosus (SLE) represent important causes of immune-mediated TIN (2, 4, 5). Among immune-mediated diseases, SS is the most common condition associated with TIN (6, 7). Here, we report a pediatric case of TIN initially presenting with recurrent UTI-like symptoms despite negative urine cultures, with subsequent evaluation suggesting an autoimmune-associated phenotype with predominant Sjögren features. This case highlights the diagnostic challenges of autoimmune-associated TIN in children.

## 2 Materials and methods

This study was approved by the Ethics Committee of Qingdao University Affiliated Hospital. Written informed consent was obtained from the guardians of the patient prior to data collection.

We retrospectively collected and reviewed the patient's clinical data, including demographic characteristics, clinical manifestations, laboratory findings, imaging studies, renal pathological findings, treatment, and follow-up data. The 2019 European League Against Rheumatism (EULAR)/American College of Rheumatology (ACR) classification criteria were applied to evaluate SLE-related features. The 2016 ACR/EULAR classification criteria for primary Sjögren syndrome and the 1999 Czech-proposed diagnostic criteria for juvenile primary Sjögren syndrome were applied to assess Sjögren-related manifestations. The 1999 Czech criteria were considered a supportive framework for evaluating juvenile Sjögren syndrome–related features rather than a definitive diagnostic standard. Final diagnostic interpretation was based on comprehensive clinical, immunological, pathological, and follow-up findings.

## 3 Case presentation

**Figure 1:**
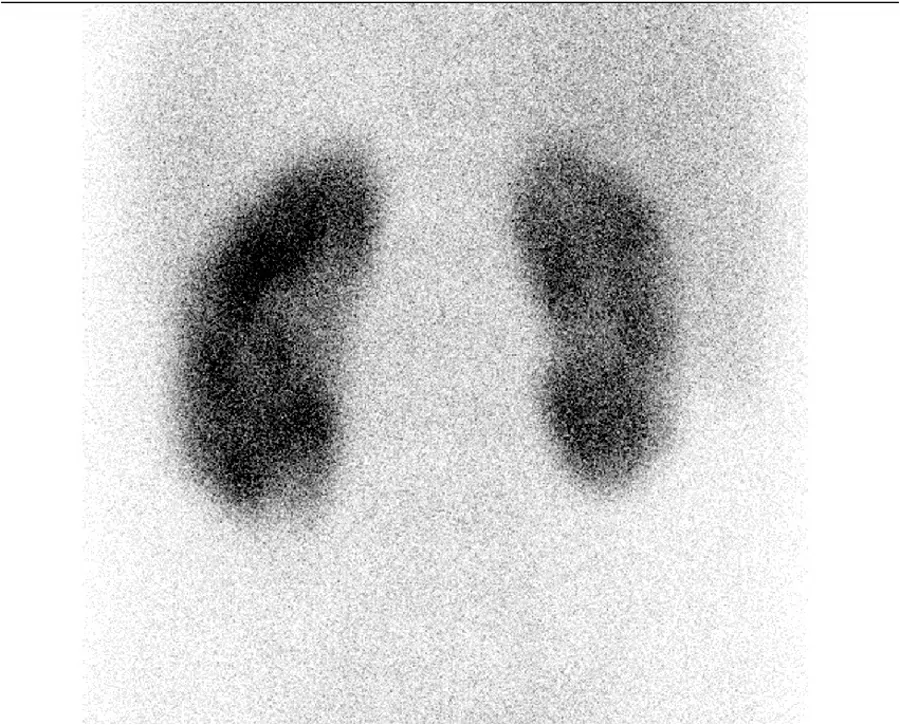
Renal static scintigraphy findings. Both kidneys are in normal position with regular contours. The right kidney is normal in size, while the left kidney shows a slight increase in size. Radiotracer distribution within both kidneys is heterogeneous, with multiple areas of decreased tracer uptake. A markedly reduced tracer distribution is noted in the left renal pelvic region. Impression: Bilateral pyelonephritis is highly suspected. Concomitant renal cortical scarring cannot be excluded, and left renal pelvic hydronephrosis is present.

A 5-year-old boy was first admitted to our department on 9 August 2022. The patient's parents were non-consanguineous, and there was no reported family history of autoimmune diseases, renal disorders, recurrent infections, or hereditary conditions. Eight months prior to admission, he developed recurrent urinary frequency without obvious precipitating factors or fever. He had visited an external hospital multiple times, where urinalyses showed white blood cell (WBC) counts ranging from 74.2 to 427.3/μL with negative urine cultures. Blood tests showed hemoglobin (Hb) 85–110 g/L and C-reactive protein (CRP) 25.70–88.8 mg/L. An ultrasound examination indicated left hydronephrosis and roughening of the bladder mucosa. Anti-infective treatment yielded only short-term efficacy, and urinary frequency recurred. Upon admission, a physical examination revealed a temperature of 36.6 °C, heart rate 96 beats per min, a blood pressure of 96/54 mmHg, a height of 110 cm (10th–25th percentile), and a weight of 16.5 kg (3rd–10th percentile). Cardiopulmonary and abdominal examinations were unremarkable. The prepuce was redundant; the urethral meatus showed no erythema, swelling, or secretion. There was no percussion tenderness in the bilateral renal regions and no edema in the lower extremities. Blood tests performed after admission revealed mild anemia (Hb: 105 g/L) and elevated inflammatory markers (CRP: 59.40 mg/L) and urinalysis revealed significant pyuria (WBC: 365.30/μL) and low urine specific gravity (1.009). Other laboratory findings are summarized in Table 1. Two consecutive urine cultures were negative. A urinary system ultrasound showed mild separation and dilation of the left renal sinus (widest part 0.7 cm) and mild left hydronephrosis. Renal static scintigraphy (Figure 1) suggested bilateral pyelonephritis, with unexcluded renal cortical scarring, and left hydronephrosis. Retrograde cystography showed no obvious reflux. Contrast-enhanced urinary CT with three-dimensional reconstruction confirmed mild left hydronephrosis. Renal dynamic scintigraphy showed that high probability of left upper urinary tract obstruction, right hydronephrosis and right kidney shrinkage, and impaired glomerular filtration function bilaterally (126.74 mL/min; left: 70.64 mL/min, right: 56.00 mL/min). Given the recurrent urinary frequency, persistent pyuria, imaging findings suggestive of pyelonephritis, and transient improvement after previous antibiotic treatment, UTI was initially suspected. The patient received sequential empirical anti-infective therapy with piperacillin–tazobactam, cefoperazone–sulbactam, meropenem, and linezolid. During treatment, he developed fever (peak temperature 38.8 °C), whereas pyuria persisted and repeated urine cultures remained negative. Therefore, non-infectious causes were subsequently investigated.

**Table 1:** Laboratory findings at the initial admission.

Parameter	Laboratory values	Reference range	Parameter	Laboratory values	Reference range
1. Complete blood count			D-Bil (μmol/L)	5.10	0–8.00
WBC (×10^9^/L)	9.41	4.00–10.00	ALT (U/L)	50.00	9.00–50.00
Neutrophils (×10^9^/L)	5.36	1.80–6.30	AST (U/L)	62.00*	15.00–40.00
Lymphocyte (×10^9^/L)	2.98	1.30–4.50	GGT (U/L)	75.00*	10.00–60.00
RBC (×10^12^/L)	4.22	3.75–5.5	ALP (U/L)	256.00*	45.00–125.00
Hb (g/L)	105*	110–145	7. Hemolysis parameters		
MCV (fL)	77.60	76.00–90.00	DAT	Weakly positive	Negative
Platelet (×10^9^/L)	293	125–350	Ret count (×10^12^/L)	0.0924*	0.025–0.075
2. Urinalysis			Ret (%)	2.11*	0.50–1.50
U-pH	6.5	4.6–8.0	LDH (U/L)	229.0	120.0–250.0
SG	1.009*	1.010–1.025	I-Bil (μmol/L)	6.49	
U-Glu	Negative	Negative	8. Immune parameters		
U-WBC (/μL)	365.3*	0–9.0	8.1 Autoantibodies		
U-RBC (/μL)	4.6	0–5.0	ANA	1:1,00,00^a^	<1:100
BLD	Negative	Negative	Anti-dsDNA	Positive(++)^b^	Negative
U-Pro	Negative	Negative		Positive^c^	Negative
24 h U-Pro (g/24 h)	0.11	<0.15	Anti-Ro/SSA	Negative	Negative
Albumin fraction (%)	100		Anti-La/SSB	Negative	Negative
β2-MG fraction (%)	0		RF (IU/mL)	84.30*	0–20.00
α1-MG fraction (%)	0		Anti-CCP (U/mL)	<7.00	0–17.00
24 h U-Na (g/24 h)	3.12	3.00–5.00	p-ANCA	Positive	Negative
24 h U-K (g/24 h)	0.64*	2.00–4.00	c-ANCA	Negative	Negative
24 h U-Cl (g/24 h)	3.67*	6.00–9.00	PR3-ANCA	Positive	Negative
24 h U-Ca (g/24 h)	0.028*	0.10–0.30	MPO-ANCA	Negative	Negative
3. Inflammatory marker			aCL IgG (GPL U/mL)	6.88	0–10.00
CRP (mg/L)	59.40*	0–5.00	aCL IgM (MPL U/mL)	2.27	0–10.00
ESR (mm/h)	62*	0–15.00	Anti-β2GPI IgG (AU/mL)	9.98	0–20.00
PCT (ng/mL)	0.243*	<0.050	8.2 Complement and Ig		
4. Renal function			C3 (g/L)	1.18	0.9–1.8
Scr (μmol/L)	43.00	31.00–132.00	C4 (g/L)	0.363	0.1–0.4
BUN (mmol/L)	5.14	3.10–8.00	IgG (g/L)	20.90*	7.00–16.00
Cys C (mg/L)	0.96	0.30–1.20	IgM (g/L)	1.21	0.40–2.30
SUA (μmol/L)	252.00	89.20–416.00	IgA (g/L)	0.97	0.70–4.00
5. Serum electrolytes			IgE (IU/mL)	75.69	0–90.00
Serum Na (mmol/L)	141.7	137.0–147.0	IgG4 (g/L)	0.93	0.013–1.446
Serum K (mmol/L)	4.01	3.50–5.30	9. Infection screening		
Serum CL (mmol/L)	105.6	99.00–110.00	Urine culture		
Serum Ca (mmol/L)	2.32	2.11–2.52	Aug 11, 2022	Negative	Negative
Serum P (mmol/L)	1.54*	0.85–1.51	Aug 21, 2022	Negative	Negative
Serum Mg (mmol/L)	1.04*	0.75–1.02	G/GM test	Negative	Negative
S-total CO_2_ (mmol/L)	23.88	23.00–31.00	T-SPOT.TB	Negative	Negative
AG (mmol/L)	12.20	8.00–16.00	HBsAg (IU/mL)	0.00	<0.05
6. Hepatic function			Anti-HCV IgG (S/CO)	0.03	<1.00
Alb (g/L)	37.27*	40.0–55.0	Anti-TP (S/CO)	0.05	<1.00
T-Bil (μmol/L)	11.59	3.00–22.00	Anti-HIV-1/2 (S/CO)	0.1	<1.00

BLD, blood; GPL, IgG phospholipid units; MPL, IgM phospholipid units; MCV, mean corpuscular volume; SG, specific gravity; U-WBC, urinary white blood cell count; U-RBC, urinary red blood cell count; U-Pro, urinary protein; 24 h U-Pro, 24-hour urinary protein; β2-MG, β2-microglobulin; α1-MG, α1-microglobulin; U-Na, urinary sodium; U-K, urinary potassium; U-Cl, urinary chloride; U-Ca, urinary calcium; CRP, C-reactive protein; ESR, erythrocyte sedimentation rate; PCT, procalcitonin; Scr, serum creatinine; BUN, blood urea nitrogen; Cys C, cystatin C; SUA, serum uric acid; S-total CO_2_, serum total carbon dioxide; AG, serum anion gap; D-Bil, direct bilirubin; ALT, alanine aminotransferase; AST, aspartate aminotransferase; GGT, gamma-glutamyl transferase; ALP, alkaline phosphatase; DAT, direct antiglobulin test; Ret, reticulocyte count; LDH, lactate dehydrogenase; I-Bil, indirect bilirubin; ANA, antinuclear antibody; anti-dsDNA, anti-double-stranded DNA antibody; anti-Ro/SSA, anti-Sjögren syndrome–related antigen A antibody; anti-La/SSB, anti-Sjögren syndrome–related antigen B antibody; RF, rheumatoid factor; anti-CCP, anticyclic citrullinated peptide antibody; ANCA, antineutrophil cytoplasmic antibody; PR3-ANCA, proteinase 3 antineutrophil cytoplasmic antibody; MPO-ANCA, myeloperoxidase antineutrophil cytoplasmic antibody; aCL, anticardiolipin antibody; anti-β2GPI, anti-beta-2-glycoprotein I antibody; Ig, immunoglobulin; C3, complement component 3; C4, complement component 4; G/GM, glucan/galactomannan; T-SPOT.TB, T-cell spot test for tuberculosis; HBsAg, hepatitis B surface antigen; anti-HCV, hepatitis C virus antibody; anti-TP, Treponema pallidum antibody; anti-HIV-1/2, human immunodeficiency virus type 1/2 antibody.

Reference ranges were based on age- and sex-adjusted intervals provided by the hospital laboratory. Abnormal values are marked with an asterisk.

a The ANA test was performed by indirect immunofluorescence on HEp-2 cells. The staining pattern was homogeneous, with a titer of 1:10,000.

b The anti-dsDNA antibody was detected as positive (++) by immunoblotting.

c The anti-dsDNA antibody was detected as positive by CLIFT.

**Figure 2:**
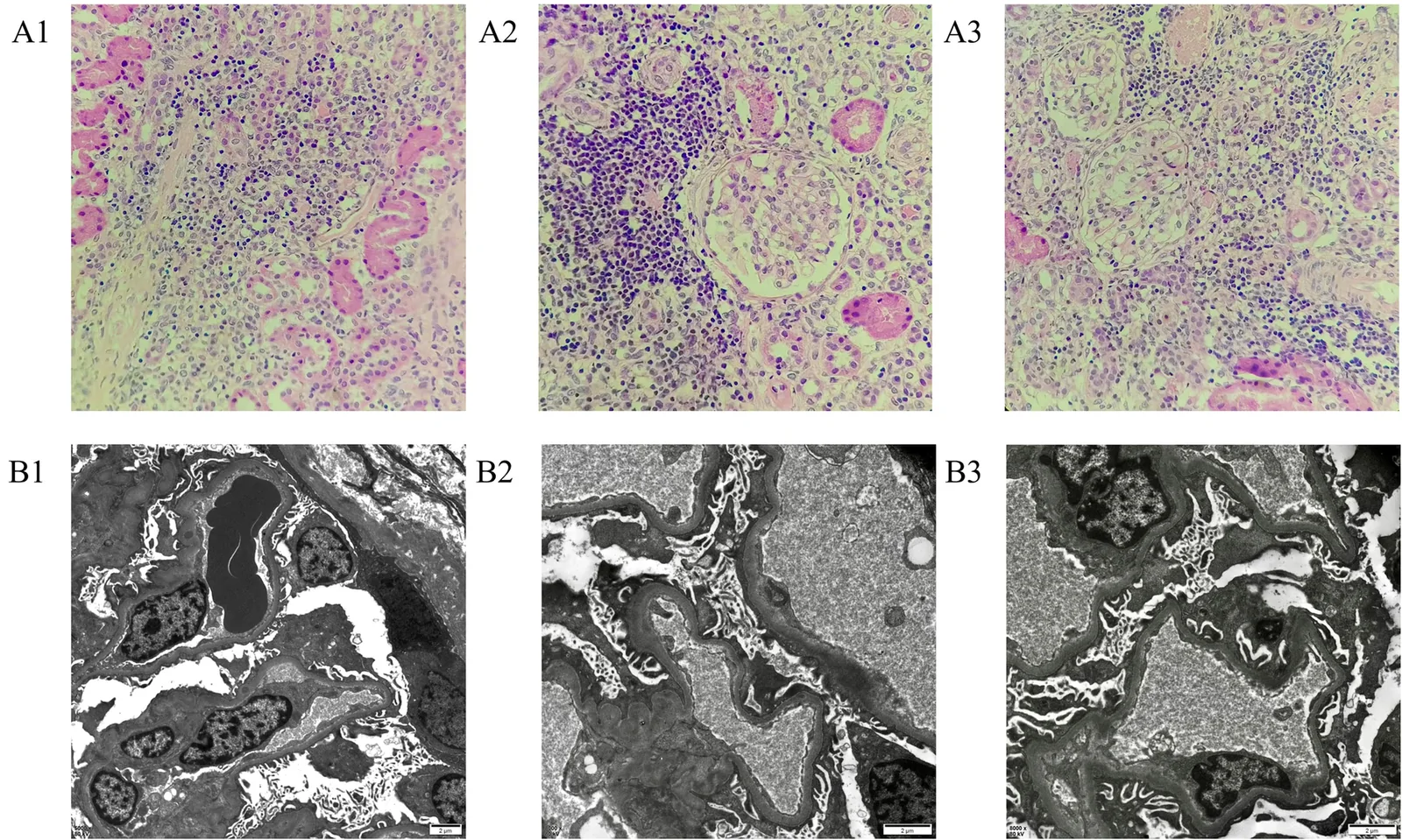
Histopathological findings of a kidney biopsy. **(A1–A3)** Light microscopy demonstrated severe acute tubulointerstitial lesions with chronic changes, diffuse flattening of tubular epithelial cells, loss of brush borders, mild tubular atrophy, basement membrane thickening, tubulitis, and a few protein casts; diffuse interstitial infiltration of mononuclear cells, many plasma cells, a few eosinophils, and occasional neutrophils. Immunofluorescence: IgG, IgA, IgM, C3, C1q, *κ*-chain, and *λ*-chain were all negative. Immunohistochemistry: abundant IgG1 positive plasma cells, rare IgG2 and IgG3 positive plasma cells, few IgG4 positive plasma cells, PLA2R negative, Congo red negative. **(B1–B3)** Electron microscopy showing vacuolar degeneration of tubular epithelial cells, focal tubular atrophy, and interstitial inflammatory cell infiltration. Mild mesangial cell and matrix proliferation and extensive podocyte foot process effacement were observed. No electron-dense deposits were identified.

Further history-taking revealed polydipsia and polyuria starting 6 months prior, accompanied by nocturia and nocturnal enuresis. A review of previous urinalyses showed specific gravity consistently ranging between 1.001 and 1.010, raising a high suspicion for TIN. Further investigations revealed a high antinuclear antibody (ANA) titer of 1:10,000 by indirect immunofluorescence on HEp-2 cells, showing a homogeneous pattern. Immunoblot-based extractable nuclear antigen (ENA) profiling showed positive anticentromere antibody and anti-double-stranded DNA (anti-dsDNA) antibody (++), whereas the results of anti-Sjögren syndrome–related antigen A antibody (anti-SSA/Ro) and anti-Sjögren syndrome-–related antigen B antibody (anti-SSB/La) antibody tests were negative. Anti-dsDNA antibody was further confirmed to be positive by *Crithidia luciliae* indirect immunofluorescence testing (CLIFT). Rheumatoid factor (RF) was elevated, and both p-antineutrophil cytoplasmic antibody (ANCA) and proteinase 3 antineutrophil cytoplasmic antibody (PR3-ANCA) were positive. The result of the direct antiglobulin (Coombs) test was weakly positive. Complement (C), antiphospholipid antibodies, and thyroid function were normal. A salivary gland ultrasound showed abnormal echogenicity in the submandibular gland. An ophthalmologic examination showed no uveitis; a tear secretion test and tear film breakup time could not be performed because of non-cooperation from the patient. A lip biopsy was initially deferred because of poor cooperation. To clarify the diagnosis, a renal biopsy was performed. Pathology (Figure 2): A histopathological examination revealed severe tubulointerstitial inflammation with chronic changes and mild glomerular involvement. Immunofluorescence staining was negative for immunoglobulins and complement components, and electron microscopy showed no electron-dense deposits.

Based on the child's polydipsia, polyuria, persistently low urine specific gravity, and renal biopsy findings, TIN was diagnosed. Given the persistent sterile pyuria, a lack of a sustained response to antibiotics, and multiple immunological abnormalities suggestive of systemic autoimmunity, an immune-mediated etiology was considered, following which glucocorticoid therapy was initiated. The patient's temperature normalized, polydipsia and polyuria gradually resolved, urine WBC became negative, urine specific gravity rose to 1.013, and Hb rose to 113–130 g/L. At the second admission for follow-up evaluation and further investigation on 5 October 2022, the patient's polydipsia and polyuria had resolved, and urinary leukocytes remained negative. ANA remained positive (homogeneous pattern, 1:3,200), anti-dsDNA antibody was weakly positive, serum C3 had decreased to 0.76 g/L (reference range: 0.90–1.80 g/L), with a normal C4 level, and the result of the direct antiglobulin (Coombs) test remained weakly positive. Given the unusually early onset and atypical autoimmune phenotype, singleton whole-exome sequencing was subsequently performed to investigate possible monogenic autoimmune or immune-dysregulatory disorders. No pathogenic or likely pathogenic variants clearly associated with the patient's phenotype were identified. To further evaluate the possibility of juvenile Sjögren disease, a labial gland biopsy was performed on 10 October 2022. The pathology showed chronic inflammation with focal fibrous hyperplasia of the interstitium and occasional lymphocyte infiltration, without focal lymphocytic sialadenitis meeting a focus score ≥1. Several SLE-associated features were identified. However, even after the subsequent mild decrease in serum C3 was taken into account, the patient did not meet the 2019 EULAR/ACR classification threshold because certain potentially contributing items, particularly autoimmune hemolytic anemia, were not sufficiently supported by the available clinical and laboratory evidence. According to the proposed 1999 Czech criteria for juvenile primary Sjögren syndrome, the patient fulfilled the criteria based on fever, immunological abnormalities including positive ANA and RF, elevated erythrocyte sedimentation rate (ESR), and hypergammaglobulinemia. Together with the tubulointerstitial pattern of renal involvement and abnormal salivary gland ultrasonography, these findings favored juvenile Sjögren syndrome as the more likely autoimmune association. The case was therefore interpreted as autoimmune-associated TIN with predominant Sjögren features, while definite SLE or an overlap phenotype could not be excluded.

Immunosuppressive therapy was initiated with intravenous methylprednisolone (15 mg twice daily, approximately 2 mg/kg/day) from 7 to 8 September 2022, followed by 12.5 mg twice daily from 9 to 14 September 2022. Oral prednisone (12.5 mg twice daily) was subsequently administered from 15 September 2022, with gradual tapering during outpatient follow-up. Mycophenolate mofetil (MMF) was added on 12 October 2022, at 0.25 g twice daily (approximately 30 mg/kg/day), increased to 0.375 g twice daily from 14 October 2022 and maintained until July 2024. A follow-up on 30 January 2023 showed that ANA remained positive (homogeneous pattern, 1:1,000), whereas the results of the direct antiglobulin (Coombs) test and anti-dsDNA antibody test were negative, and C3, C4, and RF were within the normal range. Prednisone was gradually tapered and reduced to 1.25 mg every other day on 23 May 2023. Because of recurrent urinary leukocytosis suggestive of possible disease activity, prednisone was increased to 5 mg daily from 23 July 2023. In July 2024, the patient developed recurrent urinary frequency, increased urinary leukocytes, elevated erythrocyte sedimentation rate, and elevated transaminase levels, suggesting possible disease activity accompanied by liver injury. As MMF-induced liver injury could not be excluded, the MMF dose was subsequently reduced to 0.25 g twice daily on 22 July 2024. Given the suspected disease activity and the need for intensified immunosuppression in the context of elevated transaminase levels, rituximab was initiated at 375 mg/m^2^ per dose on 8 September 2024, 22 April 2025, and 25 November 2025. Given the persistent elevation of transaminase levels, a liver biopsy was performed on 16 October 2024, and the findings suggested drug-induced hepatitis. Prednisone was temporarily increased to 20 mg twice daily (approximately 2 mg/kg/day), and MMF was discontinued on 29 October 2024. Transaminase levels gradually normalized thereafter. Prednisone was subsequently tapered during regular outpatient follow-up and was completely discontinued on 21 July 2026.

## 4 Discussion

TIN represents a non-specific category of renal diseases primarily characterized by inflammatory injury to the renal tubules and interstitium. Its etiology is complex and includes secondary causes such as drug toxicity, infections, autoimmune reactions, and systemic diseases (3, 8). The clinical presentation is variable, often manifesting as an acute or subacute onset with non-specific systemic symptoms, including fever, fatigue, anorexia, nausea, and vomiting (9). Because of impaired tubular function, patients may exhibit polyuria or oliguria, hyposthenuria (low urine specific gravity), mild proteinuria, glycosuria, leukocyturia, and low-molecular-weight proteinuria (10). Although some pediatric patients may exhibit mild hematuria or hypertension, these findings are generally less prominent than in glomerular diseases (11). Histologically, TIN is characterized by tubular epithelial injury, tubulitis, and inflammatory infiltration of the renal interstitium (12). Tubular epithelial injury and leukocyte infiltration may lead to sterile pyuria. It has been reported that the incidence of sterile pyuria in TIN ranges from 30% to 82% (3, 10, 13). Because pyuria is clinically regarded as a hallmark of UTI and the presentations are highly similar, TIN is frequently misdiagnosed as UTI in clinical practice. In the case of the patient in this study, the renal manifestations were characterized by tubular dysfunction and persistent pyuria. Impaired urinary concentrating ability was evidenced by persistent polyuria, polydipsia, and hyposthenuria. Urinary pH was 6.5, glycosuria was absent, and serum total CO_2_ and anion gap were within the normal range, without biochemical evidence of overt metabolic acidosis. Urinary β2-microglobulin and α1-microglobulin levels were not elevated, indicating no evidence of significant low-molecular-weight proteinuria. Repeatedly negative urine cultures were consistent with sterile pyuria, while a renal biopsy confirmed tubulointerstitial inflammation. Together, these findings supported the diagnosis of TIN with predominant tubular dysfunction. In children, drug-induced TIN is among the most frequently reported causes and is commonly associated with antibiotics, non-steroidal anti-inflammatory drugs, and other medications. Infectious causes, including bacterial and viral infections, may mimic immune-mediated TIN because of overlapping manifestations such as fever, pyuria, and abnormal renal imaging findings. Autoimmune and systemic inflammatory diseases, including SS, SLE, and tubulointerstitial nephritis and uveitis (TINU) syndrome, are important causes of immune-mediated TIN. In patients with very early onset and atypical autoimmune manifestations, or persistent inflammation, monogenic immune dysregulation disorders, including autoimmune regulator (AIRE)-related disorders such as autoimmune polyendocrinopathy-candidiasis-ectodermal dystrophy (APECED), should also be considered. APECED-associated renal manifestations have been increasingly recognized and may include tubulointerstitial nephritis, although they usually occur in the context of broader autoimmune and endocrine manifestations (14).

In this patient, infectious pyelonephritis was initially suspected because of recurrent urinary frequency, pyuria, hydronephrosis, and abnormal renal imaging. However, repeated urine cultures remained negative, no causative pathogen was identified, and pyuria and tubular dysfunction persisted despite broad-spectrum antibiotic therapy. A renal biopsy showed predominantly mononuclear and plasma-cell interstitial infiltration without features of acute suppurative inflammation, making active infection less likely. Drug-induced TIN was also considered because of repeated antibiotic exposure. However, the urinary symptoms and persistent hyposthenuria preceded the documented broad-spectrum antibiotic regimens used during the current admission and could not be temporally attributed to a specific drug, making these agents unlikely to be the initiating cause. Urological abnormalities were considered because of hydronephrosis and possible upper urinary tract obstruction on scintigraphy. Nevertheless, the mild structural abnormalities did not adequately explain the impaired urinary concentrating ability, persistent sterile pyuria, systemic immunological abnormalities, or biopsy-proven inflammatory TIN. Given the unusually early onset and atypical autoimmune phenotype, singleton whole-exome sequencing was performed to investigate monogenic autoimmune or immune-dysregulatory disorders. No pathogenic or likely pathogenic variant clearly explaining the phenotype was identified. The patient also lacked the characteristic endocrine, candidal, and ectodermal manifestations of APECED. Although a genetic cause could not be completely excluded, the overall serological, glandular, and renal findings made an acquired autoimmune-associated etiology the most plausible explanation at that stage. Other autoimmune disorders were considered because of anticentromere antibody and p-ANCA/PR3-ANCA positivity. However, the absence of systemic sclerosis features and pauci-immune necrotizing or crescentic glomerulonephritis on renal biopsy made the diagnosis of systemic sclerosis and ANCA-associated vasculitis unlikely.

SS is a rare chronic systemic autoimmune disease primarily affecting exocrine glands (e.g., salivary and lacrimal glands), leading to symptoms such as xerostomia and keratoconjunctivitis sicca, although it may affect multiple organs (15). Compared with adults, pediatric SS patients often present with insidious typical symptoms (dry mouth/eyes). Parotitis, serological abnormalities, renal and neurological symptoms, and non-specific symptoms (fever, arthralgia) are often early manifestations, leading to misdiagnosis or underdiagnosis (16). Renal involvement is uncommon in adult SS (<10%) but appears to be relatively more frequent in pediatric patients, with reported prevalence ranging from approximately 5%–20%. It primarily manifests as glomerulonephritis caused by immune complex deposition or TIN caused by direct lymphocytic infiltration, with TIN being more common. Clinical features typically include distal renal tubular acidosis (dRTA), hypokalemia, and alkaline urine (2, 6, 17). The pathological hallmark of SS-associated TIN is interstitial infiltration by mononuclear cells, predominantly CD4^+^ T cells and plasma cells, accompanied by varying degrees of tubular atrophy and interstitial fibrosis. Because of the lack of immune complex deposition, glomerular lesions in TIN are generally mild, and proteinuria is usually mild (<1 g/24 h), while renal concentrating defects, dRTA, hypokalemia, and nephrogenic diabetes insipidus are more common. Approximately 40% of patients with TIN present with unexplained electrolyte disturbances or nocturia (18, 19). Evidence supporting autoimmune-associated TIN with predominant Sjögren features in our patient includes: (1) Clinical signs of polydipsia, polyuria, and hyposthenuria suggesting renal concentrating defects and tubular dysfunction without glomerular evidence; (2) Salivary gland ultrasonography showed abnormal glandular echogenicity, and a labial gland biopsy revealed chronic inflammatory changes, suggesting possible glandular involvement; (3) Renal pathology confirmed tubulointerstitial nephritis and lacked features suggestive of immune complex–mediated glomerular disease; (4) Immunological abnormalities common in pediatric SS, such as positive ANA, elevated RF, elevated ESR, and hypergammaglobulinemia. However, the diagnosis of juvenile SS remained challenging in this patient. Several features were atypical for classical SS: (1) Classical sicca symptoms were absent, and there was no history of parotid enlargement or recurrent parotitis, which are common early manifestations in pediatric SS; (2) The results of anti-SSA/Ro and anti-SSB/La antibody tests were negative; (3) A labial gland biopsy performed after corticosteroid therapy showed chronic inflammation with limited lymphocytic infiltration rather than typical focal lymphocytic sialadenitis. Because corticosteroids may influence inflammatory activity and lymphocytic infiltration, the negative histological findings should be interpreted cautiously. Although the patient did not fulfill the 2016 ACR/EULAR classification criteria for SS because of the absence of anti-SSA/Ro antibodies, a lack of a diagnostic labial salivary gland biopsy finding, and incomplete objective ocular assessments, these criteria were primarily developed for adult SS and may not fully capture the heterogeneous manifestations of juvenile disease. The 1999 Czech-proposed diagnostic criteria for juvenile primary SS incorporate systemic manifestations and immunological abnormalities. Therefore, we used these criteria as a supportive framework to evaluate the possibility of juvenile SS in this patient, given their emphasis on systemic manifestations and immunological abnormalities that are frequently observed in pediatric disease. Based on the predominant tubulointerstitial renal involvement, salivary gland abnormalities, and systemic autoimmune features, juvenile SS-related TIN was considered the most plausible explanation. The detailed evaluation of Sjögren syndrome–related features according to the 2016 ACR/EULAR classification criteria and the 1999 Czech-proposed criteria is summarized in Tables 2, 3, respectively.

**Table 2:** Diagnostic evaluation for juvenile Sjögren syndrome and SLE (2016 ACR/EULAR Classification Criteria for Adult Sjögren Syndrome).

Criterion	Weight	Evidence in this patient	Interpretation
Anti-SSA/Ro positivity	3	Negative	Not met
Labial salivary gland biopsy (focus score ≥1)	3	Not demonstrated	Not met
Ocular staining score ≥5	1	Not performed	Unavailable
Schirmer test ≤5 mm/5 min	1	Not performed	Unavailable
Unstimulated salivary flow ≤0.1 mL/min	1	Not performed	Unavailable
Overall assessment	≥4 required	Total score insufficient	Does not fulfill 2016 classification criteria

This table summarizes the application of the relevant classification/proposed diagnostic criteria and highlights the distinction between disease classification and clinical diagnosis. Failure to fulfill the adult classification criteria does not exclude juvenile Sjögren syndrome.

**Table 3:** Diagnostic evaluation for juvenile Sjögren syndrome and SLE (application of the 1999 Czech-Proposed Diagnostic Criteria for Juvenile Primary Sjögren Syndrome).

Criterion category	Evidence required/clinical feature	Evidence in this patient	Assessment
Systemic manifestations	Fever or other systemic manifestations	Fever developed during hospitalization (peak temperature 38.8 °C)	Fulfilled
Immunological abnormalities	Positive ANA and/or RF	ANA positive (1:10,000) RF elevated (84.30 IU/mL)	Fulfilled
Elevated inflammatory markers	Elevated ESR	ESR 62 mm/h	Fulfilled
Hypergammaglobulinemia	Increased serum immunoglobulin levels, particularly IgG	IgG elevated (20.90 g/L)	Fulfilled
Salivary gland involvement	Evidence of salivary gland abnormality	Salivary gland ultrasonography showed abnormal echogenicity of the submandibular gland; a labial gland biopsy showed chronic inflammation with focal interstitial fibrous hyperplasia and occasional lymphocyte infiltration	Supportive
Anti-SSA/Ro or anti-SSB/La antibodies	Positive anti-SSA/Ro and/or anti-SSB/La antibodies	The results of anti-SSA/Ro and anti-SSB/La antibody tests were negative	Not fulfilled
Ocular involvement	Objective evidence of keratoconjunctivitis sicca (e.g., Schirmer test)	Schirmer test and tear film breakup time could not be performed because of poor cooperation	Not assessed
Renal involvement	Tubulointerstitial nephritis or renal tubular dysfunction	A renal biopsy confirmed tubulointerstitial nephritis	Supportive

The 1999 Czech criteria are proposed diagnostic criteria for juvenile primary Sjögren syndrome rather than internationally validated classification criteria. In this study, this table is not intended to establish a definitive diagnosis of juvenile Sjögren syndrome but is used as a supportive framework for clinical assessment and comprehensive evaluation of the patient's autoimmune phenotype.

To date, only a limited number of pediatric cases of Sjögren-associated TIN have been reported. Among these, the case reported by Bouchalova et al. is particularly relevant because a 15-year-old boy presented with fever, renal imaging abnormalities, and persistent sterile pyuria, with infection repeatedly excluded. Although he had negative ANA, RF, anti-Ro/SSA, and anti-La/SSB antibodies, objective ocular involvement and salivary gland biopsy findings supported the diagnosis of juvenile primary SS (20). Similar to that patient, our patient presented with culture-negative urinary abnormalities and predominant tubulointerstitial renal involvement rather than typical glomerular disease. However, our patient differed by a much younger age at onset, the absence of typical sicca manifestations, incomplete glandular pathological evidence, and the presence of lupus-associated serological abnormalities, which increased the complexity of distinguishing Sjögren-associated autoimmune TIN from an evolving lupus-spectrum autoimmune phenotype.

In pediatric patients with childhood-onset SLE (cSLE), renal involvement is one of the most common and prognostically significant organ manifestations, affecting approximately 50%–75% of patients during the disease course (21). Lupus nephritis (LN) in children primarily manifests as glomerular lesions, predominantly proliferative types, with Class IV being the most common according to the ISN/RPS classification (22–24). In addition to typical glomerular lesions, some cases may be complicated by vascular lesions, interstitial inflammation, or fibrosis (25). Tubulointerstitial lesions are usually associated with severe glomerular pathology. In one pediatric cohort, tubulointerstitial lesions were identified in 38.81% of patients with lupus nephritis and were more frequent in proliferative than in non-proliferative disease (46.15% vs. 13.33%) (26). However, isolated tubulointerstitial injury—so-called predominant tubulointerstitial lupus nephritis—in the presence of minimal glomerular abnormalities is rare (27). Against this background, SLE was reasonably considered during the diagnostic workup because of several lupus-associated serological abnormalities. However, the diagnosis remained uncertain for the following reasons: (1) the patient lacked typical systemic manifestations of SLE, including cutaneous involvement, serositis, neurological manifestations, and significant hematological abnormalities; (2) there was no hematuria or proteinuria, and a renal biopsy showed predominant tubulointerstitial inflammation with only mild glomerular changes, negative immunofluorescence, and no electron-dense deposits, which contrasted with typical immune complex–mediated lupus nephritis; (3) some potential classification items, particularly autoimmune hemolytic anemia and fever, were not sufficiently supported or could not be confidently attributed to SLE. The detailed application of the 2019 EULAR/ACR classification criteria is summarized in Table 4.

**Table 4:** Diagnostic evaluation for juvenile Sjögren syndrome and SLE (2019 EULAR/ACR Classification Criteria for SLE).

Domain/item	Criterion	Criterion weight	Evidence in this patient	Points	Interpretation
Entry criterion	ANA ≥ 1:80	Entry criterion	ANA positive by HEp-2 IIF (1:10,000)^a^		Met
Fever	>38.3 °C	2	Developed fever during hospitalization	0	Attribution to SLE uncertain
Autoimmune hemolytic anemia	Evidence of immune-mediated hemolysis	4	Weak DAT, mild anemia, and insufficient hemolysis evidence	0	Not sufficiently supported
Complement	Low C3 or C4	3	C3 0.76 g/L (second admission), C4 normal	3	Supports classification
SLE-specific antibodies	Anti-dsDNA	6	Positive by CLIFT^b^ @ Positive (++) by IBT^c^	6	Supports classification
Overall assessment	Classification threshold ≥10				Classification criteria do not establish a definitive diagnosis

a HEp-2 IIF, HEp-2 cell indirect immunofluorescence assay.

b CLIFT, *C. luciliae* indirect immunofluorescence test.

c IBT, Immunoblotting test.

Although SLE and Sjögren syndrome are distinct systemic autoimmune diseases, they may share clinical and immunological features, and Sjögren manifestations may coexist with or occasionally precede SLE. In this patient, SLE remained a reasonable differential diagnosis but was insufficiently supported as a definitive diagnosis. Long-term follow-up is therefore warranted to monitor for possible evolution toward definite SLE or an overlap phenotype.

This case highlights the diagnostic complexity of autoimmune-associated TIN in children, particularly when presenting as recurrent, culture-negative urinary symptoms. The combination of tubulointerstitial renal injury, salivary gland abnormalities, and systemic autoimmune features favored an autoimmune phenotype with predominant Sjögren features.

Several limitations in this study should be acknowledged. First, the patient did not fulfill the 2016 ACR/EULAR classification criteria for SS because of negative anti-SSA/Ro antibodies, the absence of a diagnostic labial salivary gland biopsy finding, and incomplete objective ocular assessments; moreover, the interpretation of salivary gland histology should consider the potential influence of prior corticosteroid therapy. Second, although a renal biopsy and available laboratory findings supported TIN with tubular dysfunction, further characterization of tubular involvement was limited because additional functional assessments, such as fractional electrolyte excretion, were not performed. Third, although whole-exome sequencing did not identify pathogenic variants explaining the phenotype, unidentified genetic causes or immune dysregulation disorders could not be completely excluded. Given the evolving nature of pediatric autoimmune diseases, long-term follow-up and studying additional cases will be important to further clarify the clinical spectrum and mechanisms of autoimmune-associated TIN in childhood.

## 5 Conclusions

Autoimmune-associated TIN should be considered in children with recurrent, culture-negative urinary symptoms and persistent pyuria. This case highlights the heterogeneous presentation of juvenile autoimmune–associated TIN and the need for long-term follow-up to clarify disease evolution.

## Data Availability

The raw data supporting the conclusions of this article will be made available by the authors, without undue reservation.
